# Energy Efficiency and Spectral Efficiency Tradeoff in Massive MIMO Multicast Transmission with Statistical CSI

**DOI:** 10.3390/e22091045

**Published:** 2020-09-18

**Authors:** Bin Jiang, Bowen Ren, Yufei Huang, Tingting Chen, Li You, Wenjin Wang

**Affiliations:** 1 National Mobile Communications Research Laboratory, Southeast University, Nanjing 210096, China; renbw@seu.edu.cn (B.R.); yufei_huang@seu.edu.cn (Y.H.); ttchen@seu.edu.cn (T.C.); liyou@seu.edu.cn (L.Y.); wangwj@seu.edu.cn (W.W.); 2 Key Laboratory of Dynamic Cognitive System of Electromagnetic Spectrum Space, Ministry of Industry and Information Technology, Ministry of Industry and Information Technology, Nanjing University of Aeronautics and Astronautics, Nanjing 210016, China; 3 Purple Mountain Laboratories, Nanjing 211100, China

**Keywords:** wirelessly powered communications, energy efficiency, spectral efficiency, statistical channel state information (CSI), massive MIMO, resource allocation

## Abstract

As the core technology of 5G mobile communication systems, massive multi-input multi-output (MIMO) can dramatically enhance the energy efficiency (EE), as well as the spectral efficiency (SE), which meets the requirements of new applications. Meanwhile, physical layer multicast technology has gradually become the focus of next-generation communication technology research due to its capacity to efficiently provide wireless transmission from point to multipoint. The availability of channel state information (CSI), to a large extent, determines the performance of massive MIMO. However, because obtaining the perfect instantaneous CSI in massive MIMO is quite challenging, it is reasonable and practical to design a massive MIMO multicast transmission strategy using statistical CSI. In this paper, in order to optimize the system resource efficiency (RE) to achieve EE-SE balance, the EE-SE trade-offs in the massive MIMO multicast transmission are investigated with statistical CSI. Firstly, we formulate the eigenvectors of the RE optimization multicast covariance matrices of different user terminals in closed form, which illustrates that in the massive MIMO downlink, optimal RE multicast precoding is supposed to be done in the beam domain. On the basis of this viewpoint, the optimal RE precoding design is simplified into a resource efficient power allocation problem. Via invoking the quadratic transform, we propose an iterative power allocation algorithm, which obtains an adjustable and reasonable EE-SE tradeoff. Numerical simulation results reveal the near-optimal performance and the effectiveness of our proposed statistical CSI-assisted RE maximization in massive MIMO.

## 1. Introduction

Due to the traffic requirement of the group-oriented applications and services in 5G communication systems increasing drastically, wireless multicast transmission has obtained broad research interests [1,2] and has been embodied in the 3GPP releases [3]. In order to simultaneously serve multiple user terminals (UTs) in the same frequency/time resources, massive multiple-input multiple-output (MIMO) equips substantial antennas at the base station (BS), which can dramatically enhance the spectral efficiency (SE) over conventional MIMO systems [4,5]. Owing to massive MIMO’s capacity to shape the multicast transmission signal efficiently, thereby improving the quality of service, massive MIMO multicast is a promising candidate for future wireless communication [2].

Considerable attention from academia and industry has concentrated on energy efficiency (EE) for wireless communications in the past several years for both ecological and economic reasons [6,7]. Traditionally, SE is considered as a more significant design object than EE to obtain a higher transmission rate without considering cost. Nevertheless, even if the wireless network can reach the required high transmission rates, power consumption may increase sharply, which calls for an environmentally-friendly system design [8]. As a result, EE has appeared as a substantial performance indicator of wireless transmitting design and has drawn much more attention [6,9]. In some recent research works, EE optimization design for MIMO unicasting has been studied, e.g., [10,11,12]. Meanwhile, several previous works also investigated energy efficient multicasting transmission. For instance, in [13,14], energy efficient multicast and unicast joint transmission for the multi-cell multi-user MIMO system were investigated. Besides, coordinated energy efficient transmission for multi-cell multicasting was investigated in [15,16]. However, the EE optimal strategy may sometimes conflict with the SE optimal strategy. Therefore, it is worth investigating how to achieve a balance between SE and EE.

The quality of channel state information (CSI) is a significant factor affecting SE and EE transmission performance. The existing EE-SE tradeoff can be roughly divided into two categories. One is to jointly maximize EE and SE by introducing a tradeoff factor [14,15]. The others are to maximize EE under a certain SE requirement [16,17]. However, it can be noticed that most of the existing massive MIMO transmission design works, e.g., [13,14,15,16,17,18], are on the basis of the presumption that instantaneous CSI at the transmitter is known. However, due to the acquisition overhead, hardware defects, and other practical limitations, it is quite challenging to obtain perfect instantaneous CSI in massive MIMO [19,20]. Since statistical CSI changes more on the time scale than instantaneous CSI, which is much more efficient and accurate to obtain, it is reasonable to employ statistical CSI for designing massive MIMO multicast transmission strategies. Furthermore, as the antenna array expands, the new statistical channel characteristics shown in large-scale MIMO have the capacity to facilitate practical transmission design [21].

This paper studies the EE-SE tradeoff design for massive MIMO multicast precoding with the BS only knowing statistical CSI. In order to reach an adaptive EE-SE tradeoff, we adopt a variable environmental parameter, resource efficiency (RE) [22], and discuss RE maximization. The main contributions of our work are listed below:

- With statistical CSI, we determine the eigenvectors of the optimal multicast transmit covariance matrix in closed form to maximize the system RE, which demonstrates that in massive MIMO multicast, optimal RE multicast transmission is supposed to be performed in the beam domain. Thus, the complex matrix-valued large-dimensional multicast precoding design problem for massive MIMO is reduced to a beam domain power allocation problem with a notable reduction of the optimization variables.
- Via adopting the quadratic transform, we propose a power allocation algorithm that can obtain an adjustable and reasonable EE-SE tradeoff.
- We deduce the deterministic equivalent (DE) of the design target, which is derived from the theory of large-dimensional random matrices, to further reduce the computational complexity of the RE maximization problem.

The remainder of the paper is constructed as follows. The massive MIMO channel model is introduced in Section 2. In Section 3, we introduce the system RE and investigate the transmission approach for RE optimization with only statistical CSI. In Section 4, numerical results are drawn. Section 5 presents the conclusion.

The notations utilized in this paper are given below:

- Matrices and column vectors are represented by upper- and lower-case boldface letters, respectively, whereas italic letters represent scalars.
- ${\left[A\right]}_{m,n}$ represents the (m,n)th element of matrix A.
- X⪰0 denotes that X is a positive semidefinite matrix.
- $I_{M}$ denotes the M×M identity matrix.
- ⊙ represents the Hadamard product.
- $E\left\{.\right\}$ denotes the expectation operation.
- ∼ denotes “be distributed as”, and ≜ denotes “be defined as”.
- We use $C^{M\times N}$ to represent an M×N-dimensional complex-valued vector space and $R^{M\times N}$ to denote an M×N-dimensional real-valued vector space.
- Denote by ${\left(.\right)}^{*}$ the conjugate operation, ${\left(.\right)}^{T}$ the transpose operation, ${\left(.\right)}^{H}$ the conjugate-transpose operation, $\det\left\{.\right\}$ the determinant operation, and $\mathrm{tr}\left\{.\right\}$ the trace operation.

## 2. Massive MIMO Downlink Channel Model

Consider a single-cell massive MIMO multicast transmission system consisting of an *M*-antenna BS and *K* UTs. The BS delivers one single group multicast signal of common interest to each UT *k*, which has $N_{k}$ receive antennas.

Let $x\in C^{M\times 1}$ be the multicast signal sent to the UTs. Assume that the mean of the transmit signal is x$E\left\{x\right\}=0$ and the covariance matrix is $E\left\{xx^{H}\right\}=Q\in C^{M\times M}$, where Q represents its covariance matrix, then the received signal at UT *k* is formulated by:

(1) $$\begin{array}{cc} y_{k} & =H_{k}x+n_{k}\in C^{N_{k}\times 1}, \end{array}$$

where $H_{k}\in C^{N_{k}\times M}$ is the downlink channel matrix between the BS and UT *k* and $n_{k}∼\mathrm{CN}\left(0,\sigma^{2}I_{N_{k}}\right)$ represents the complex-valued additive Gaussian noise with $\sigma^{2}$ being the noise power.

In the paper, we apply Weichselberger’s Rayleigh fading model [23], which jointly characterizes the correlation properties from the transmitter (BS) to the receiver (UT). Specifically, the downlink channel matrix $H_{k}$ in (1) is modeled as follows:

(2) $$\begin{array}{cc} H_{k} & =U_{k}G_{k}V_{k}^{H}\in C^{N_{k}\times M}, \end{array}$$

where $U_{k}\in C^{N_{k}\times N_{k}}$ and $V_{k}\in C^{M\times M}$ are deterministic unitary matrices and $G_{k}\in C^{N_{k}\times M}$ is the downlink channel matrix in the beam domain with zero-mean independent elements [21]. The statistical CSI of $G_{k}$ can be denoted as:

(3) $$\begin{array}{cc} \Omega_{k} & =E\left\{G_{k}⊙G_{k}^{*}\right\}\in R^{N_{k}\times M}, \end{array}$$

where $\Omega_{k}$ is always referred to as the channel power matrix in the beam domain, which varies on a time scale larger than instantaneous CSI. Moreover, $\Omega_{k}$ has the property of maintaining nearly constant in a wide frequency range so that statistical CSI can be acquired in an effective and accurate way [21].

For the massive MIMO systems, the eigenvector matrix of the transmit correlation matrix from the BS to each UT tends to be equivalent to a deterministic unitary matrix V, due to the massive antenna arrays adopted at the BS, which merely relies on the array topology [21]. Then, the downlink channel matrix in the massive MIMO scenario can be well approximated as:

(4) $$\begin{array}{cc} H_{k} & \overset{M\to \infty }{=}U_{k}G_{k}V^{H}. \end{array}$$

Please note that many previous works used the above approximations, which have exhibited high accuracy in typical scenarios [21,24,25,26]. The remainder of this paper shall employ the channel model for massive MIMO in (4).

## 3. Multicast Precoding for RE Maximization

### 3.1. Problem Formulation

Assume that every UT identifies its own instantaneous CSI, and the BS can only access the statistical CSI of each UT. Then, utilizing multicast transmission covariance matrix Q, we define the rate of achievable ergodic multicast below:

(5) $$\begin{array}{cc} R_{\mathrm{mc}} & =\min_{k}\;R_{k}, \end{array}$$

where $R_{k}$ represents the achievable ergodic rate of the *k*th UT, which is formulated by:

(6) $$\begin{array}{cc} R_{k} & =E\left\{\log\det\left\{I_{N_{k}}+\frac{1}{\sigma^{2}}H_{k}QH_{k}^{H}\right\}\right\}\\ \; & \overset{\left(a\right)}{=}E\left\{\log\det\left\{I_{N_{k}}+\frac{1}{\sigma^{2}}G_{k}V^{H}QVG_{k}^{H}\right\}\right\}, \end{array}$$

where (a) inserts the system model for massive MIMO in (4) and $\det\left\{I+XY\right\}=\det\left\{I+YX\right\}$. Then, we can define the system SE of multicast transmission as:

(7) $$\begin{array}{cc} \eta_{\mathrm{SE}}=R_{\mathrm{mc}} & =\min_{k}\;R_{k}\left(\mathrm{bits}/s/\mathrm{Hz}\right). \end{array}$$

For a massive MIMO system, most of the power consumption is contributed by the BS. Therefore, in the paper, the following power consumption model [6,12] is introduced as:

(8) $$\begin{array}{c} P=\zeta \mathrm{tr}\left\{Q\right\}+MP_{c}+P_{s}, \end{array}$$

where the scaling coefficient ζ≥1 models the reciprocal of the transmit amplifier drain efficiency, $\mathrm{tr}\left\{Q\right\}$ represents the multicast transmit power, $P_{c}$ represents the constant circuit power consumption of each antenna, which is immune to the practical transmission power, and $P_{s}$ means the static power consumption of the BS. With the above system SE in (7) and the power consumption model in (8), the system EE of multicast transmission can be defined as:

(9) $$\begin{array}{c} \eta_{\mathrm{EE}}=W\frac{\eta_{\mathrm{SE}}}{P}\left(\mathrm{bits}/\mathrm{Joule}\right), \end{array}$$

where *W* is the bandwidth.

As SE and EE are essential performance indicators in the design of communication systems, it is worth investigating how to strike a balance between the two. For this reason, by maximizing a weighted sum of SE and EE to acquire an SE-EE tradeoff, we consider simultaneously optimizing EE and SE. Nevertheless, since the metric units of EE and SE, which are bits/Joule and bits/s/Hz, respectively, are inconsistent, it seems inappropriate to directly add SE and EE. Therefore, we select a system design metric named RE [22], which is determined by:

(10) $$\begin{array}{c} \eta_{\mathrm{RE}}≜\frac{\eta_{\mathrm{EE}}}{W}+\beta \frac{\eta_{\mathrm{SE}}}{P_{\mathrm{tot}}}\left(\mathrm{bits}/\mathrm{Joule}/\mathrm{Hz}\right) \end{array}$$

where β(>0) denotes the weight factor. Please note that with EE-SE balance controlled by β, the RE metric has the capacity to obtain an SE-EE tradeoff. Moreover, $\frac{1}{W}$ and $\frac{1}{P_{\mathrm{tot}}}$ are unit normalizers, where $P_{\mathrm{tot}}$ denotes the overall power budget of the BS given by:

(11) $$\begin{array}{c} P_{\mathrm{tot}}=\zeta P_{\max}+MP_{c}+P_{s}, \end{array}$$

which is akin to (8), and $P_{\max}$ represents the multicast power budget at the BS. In addition, let $\beta \frac{W}{P_{\mathrm{tot}}}≜\alpha /\left(1-\alpha \right)$; it can be observed that $\eta_{\mathrm{RE}}$ maximization is equivalent to $\left(1-\alpha \right)\eta_{\mathrm{EE}}+\alpha \eta_{\mathrm{SE}}$ maximization. Therefore, RE maximization equals acquiring the Pareto optimal solution set of the SE-EE multi-objective optimization problem [27].

In succession, we investigate the precoding design of RE maximization in multicast transmission with statistical CSI. Our goal is to find the optimal transmission covariance Q that maximizes the multicast RE in (10). Therefore, the RE maximization problem can be expressed as:

(12) $$\begin{array}{cc} P_{1}:\max_{Q}\; & \eta_{\mathrm{RE}},\\ s.t.\; & \mathrm{tr}\left\{Q\right\}\leq P_{\max},\;Q⪰0. \end{array}$$

### 3.2. Optimal Transmit Direction

Decompose the eigenvalue of transmission covariance as $Q=\Phi \Lambda \Phi^{H}$. Thus, the eigenvalues of Q are the diagonal elements of Λ, and the eigenvectors of Q are denoted by the columns of Φ. Please note that the transmit covariance matrix’s eigenvalues and eigenvectors denote the allocated powers over each direction and the directions of the transmit signals, respectively. Firstly, we study the eigenvectors of the optimal transmission covariance matrix.

**Theorem** **1.** *The optimal transmission covariance matrix $Q^{\mathrm{opt}}$ in the RE maximization problem (12) is composed by matrix V in (4), i.e.,*

(13) $$\begin{array}{c} Q^{\mathrm{opt}}=V\Lambda V^{H}, \end{array}$$

*where the columns of V are the eigenvectors of $Q^{\mathrm{opt}}$.*

**Proof.**  Note that the consumed energy $P=\zeta \mathrm{tr}\left\{Q\right\}+MP_{c}+P_{s}$ is irrelevant to the off-diagonal elements of $V^{H}QV$. Moreover, since $R_{\mathrm{mc}}$ is a concave function of $V^{H}QV$, applying a proof method analogous to that exhibited in [28], it can be proven that $V^{H}QV$ is supposed to be diagonal for maximizing $R_{\mathrm{mc}}$. In addition, only the diagonal elements of $V^{H}QV$ can determine the transmit power $\mathrm{tr}\left\{Q\right\}$. Therefore, to maximize the design target of the problem in (12), $V^{H}QV$ has to be a diagonal matrix. This is the end of the proof. □

Theorem 1 above indicates that to achieve the optimal performance of RE maximization in the beam domain transmission, the optimal RE signaling directions are supposed to coincide with the transmission correlation matrices’ eigenvectors at the BS. Adopting Theorem 1, the complex matrix-valued transmission covariance matrix design of RE optimization is reduced to a beam domain power allocation problem. Therefore, the RE optimization problem over Q in (12) is predigested into the following problem over Λ without loss of optimality:

(14) $$\begin{array}{cc} P_{2}:\max_{\Lambda }\; & {\tilde{\eta }}_{\mathrm{RE}}\left(\Lambda \right)=\frac{{\tilde{\eta }}_{\mathrm{EE}}\left(\Lambda \right)}{W}+\beta \frac{{\tilde{\eta }}_{\mathrm{SE}}\left(\Lambda \right)}{P_{\mathrm{tot}}},\\ s.t.\; & \mathrm{tr}\left\{\Lambda \right\}\leq P_{\max},\;\Lambda \;\mathrm{diagonal},\;\Lambda ⪰0, \end{array}$$

where:

(15) $$\begin{array}{cc} {\tilde{\eta }}_{\mathrm{EE}}\left(\Lambda \right) & =\frac{W{\tilde{\eta }}_{\mathrm{SE}}\left(\Lambda \right)}{\zeta \mathrm{tr}\left\{\Lambda \right\}+MP_{c}+P_{s}}, \end{array}$$

(16) $$\begin{array}{cc} {\tilde{\eta }}_{\mathrm{SE}}\left(\Lambda \right) & =R_{\mathrm{mc}}\left(\Lambda \right)≜\min_{k}\;R_{k}\left(\Lambda \right), \end{array}$$

(17) $$\begin{array}{cc} R_{k}\left(\Lambda \right)\; & ≜E\left\{\log\det\left\{I_{N_{k}}+\frac{1}{\sigma^{2}}G_{k}\Lambda G_{k}^{H}\right\}\right\}, \end{array}$$

(18) $$\begin{array}{cc} P\left(\Lambda \right) & ≜\zeta \mathrm{tr}\left\{\Lambda \right\}+MP_{c}+P_{s}. \end{array}$$

Please note that ${\tilde{\eta }}_{\mathrm{SE}}\left(\Lambda \right)$ in the objective of (14) is a concave function over Λ and $P\left(\Lambda \right)$ is a linear function over Λ. Therefore, the RE optimization problem in (14) is a multi-ratio fractional program. The quadratic transform is a classic method for solving the multi-ratio fractional program [29], so it is adopted in this paper to figure out the optimization problem. The quadratic transform is derived from Dinkelbach’s transform, but has an additional constraint that the objective function value needs to remain unchanged. It involves quadratic terms, so it is called the quadratic transform. Via invoking the quadratic transform, the RE optimization precoding problem in (14) can be figured out by solving a range of the following convex optimization problems iteratively:

(19) $$\begin{array}{cc} \Lambda^{\left(\ell +1\right)}=\underset{\Lambda }{\arg\max}\; & 2\eta^{\left(\ell \right)}\sqrt{{\tilde{\eta }}_{\mathrm{SE}}\left(\Lambda \right)}-{\left(\eta^{\left(\ell \right)}\right)}^{2}P\left(\Lambda \right)+\beta \frac{{\tilde{\eta }}_{\mathrm{SE}}\left(\Lambda \right)}{P_{\mathrm{tot}}},\\ s.t.\; & \mathrm{tr}\left\{\Lambda \right\}\leq P_{\max},\;\Lambda \;\mathrm{diagonal},\;\Lambda ⪰0, \end{array}$$

with an introduced auxiliary variable $\eta^{\left(\ell \right)}$, which is updated iteratively below:

(20) $$\begin{array}{c} \eta^{\left(\ell \right)}=\frac{{\tilde{\eta }}_{\mathrm{SE}}\left(\Lambda^{\left(\ell \right)}\right)}{P\left(\Lambda^{\left(\ell \right)}\right)}, \end{array}$$

where *ℓ* denotes the iteration parameter. It is observed that based on the quadratic transform, the above-mentioned iterative method converges to the global optimal solution of the initial optimal design problem in (14) at a convergence rate that is strictly slower than superlinear [29].

Due to the calculation of the ergodic multicast rate involving the expectation operation, the time complexity of the convex optimization problems at each iteration in (19) is yet computationally cumbersome in practice. Furthermore, we adopt the theory of the large-dimensional random matrix in [30,31] for calculating the ergodic rate’s DE at every iteration process to decrease the complexity of the Monte Carlo average in the optimization objective. By substituting DE for the rate expression, the convex optimization problem sequences in (19) become:

(21) $$\begin{array}{cc} \Lambda^{\left(\ell +1\right)}=\underset{\Lambda }{\arg\max}\; & 2\eta^{\left(\ell \right)}\sqrt{\min_{k}\;{\bar{R}}_{k}^{\left(\ell \right)}\left(\Lambda \right)}-{\left(\eta^{\left(\ell \right)}\right)}^{2}P\left(\Lambda \right)+\min_{k}\;\beta \frac{{\bar{R}}_{k}^{\left(\ell \right)}\left(\Lambda \right)}{P_{\mathrm{tot}}},\\ s.t.\; & \mathrm{tr}\left\{\Lambda \right\}\leq P_{\max},\;\Lambda \;\mathrm{diagonal},\;\Lambda ⪰0, \end{array}$$

where ${\bar{R}}_{k}^{\left(\ell \right)}\left(\Lambda \right)$ in (21) is $R_{k}\left(\Lambda \right)$’s DE in the *ℓ*th iteration as follows:

(22) $$\begin{array}{cc} {\bar{R}}_{k}^{\left(\ell \right)}\left(\Lambda \right) & =\sum_{i=0}^{M-1}\log\left\{1+{\left[\Lambda \right]}_{i,i}{\left[\Gamma_{k}^{\left(\ell \right)}\right]}_{i,i}\right\}\\ \; & \;+\sum_{j=0}^{N_{k}-1}\log\left\{{\left[{\tilde{\Phi }}_{k}^{\left(\ell \right)}\right]}_{j,j}\right\}\;-\sum_{m=0}^{N_{k}-1}{\left[{\tilde{\Gamma }}_{k}^{\left(\ell \right)}\right]}_{m,m}{\left[{\left({\tilde{\Phi }}_{k}^{\left(\ell \right)}\right)}^{-1}\right]}_{m,m}, \end{array}$$

where the DE auxiliary variables $\Gamma_{k}^{\left(\ell \right)}\in C^{M\times M}$, ${\tilde{\Gamma }}_{k}^{\left(\ell \right)}\in C^{N_{k}\times N_{k}}$, and ${\tilde{\Phi }}_{k}^{\left(\ell \right)}\in C^{N_{k}\times N_{k}}$ are given by the iterative equations below:

(23a) $$\begin{array}{cc} \Gamma_{k}^{\left(\ell \right)} & =B_{k}\left({\left({\tilde{\Phi }}_{k}^{\left(\ell \right)}\right)}^{-1}\right), \end{array}$$

(23b) $$\begin{array}{cc} {\tilde{\Gamma }}_{k}^{\left(\ell \right)} & =C_{k}\left(\Lambda^{\left(\ell \right)}{\left(I_{M}+\Lambda^{\left(\ell \right)}\Gamma_{k}^{\left(\ell \right)}\right)}^{-1}\right), \end{array}$$

(23c) $$\begin{array}{cc} {\tilde{\Phi }}_{k}^{\left(\ell \right)} & =I_{N_{k}}+{\tilde{\Gamma }}_{k}^{\left(\ell \right)}. \end{array}$$

Please note that $B_{k}\left(X\right)≜\frac{1}{\sigma^{2}}E\left\{G_{k}^{H}XG_{k}\right\}\in C^{M\times M}$ and $C_{k}\left(X\right)≜\frac{1}{\sigma^{2}}E\left\{G_{k}XG_{k}^{H}\right\}\in C^{N_{k}\times N_{k}}$ in (23) are both diagonal matrix-valued functions with the *i*th output diagonal elements as follows:

(24) $$\begin{array}{cc} {\left[B_{k}\left(X\right)\right]}_{i,i} & =\frac{1}{\sigma^{2}}\mathrm{tr}\left\{\mathrm{diag}\left\{{\left[\Omega_{k}\right]}_{:,i}\right\}X\right\}, \end{array}$$

(25) $$\begin{array}{cc} {\left[C_{k}\left(X\right)\right]}_{i,i} & =\frac{1}{\sigma^{2}}\mathrm{tr}\left\{\mathrm{diag}\left\{{\left({\left[\Omega_{k}\right]}_{i,:}\right)}^{T}\right\}X\right\}. \end{array}$$

We can efficiently calculate the DE ${\bar{R}}_{k}^{\left(\ell \right)}\left(\Lambda \right)$ with the channel statistics $\Omega_{k}$ within several iterations; there is no need to adopt the Monte Carlo approach for exhaustive averaging, thereby further reducing the time complexity of the RE maximization problem in (19).

### 3.3. Power Allocation for Multicast Transmission

It can be noted that, for massive MIMO channels, DE ${\bar{R}}_{k}^{\left(\ell \right)}\left(\Lambda \right)$ manifests as a very close approximation of $R_{k}\left(\Lambda \right)$ in typical scenarios [30,31]. Moreover, as ${\bar{R}}_{k}\left(\Lambda \right)$ in (22) is a concave function over Λ, the subproblems in (21) are convex and still solvable efficiently. Our proposed multicast power allocation algorithm for RE optimization in the beam domain adopting the quadratic transform and DE is described in Algorithm 1.
**Algorithm 1** Multicast power allocation algorithm in the beam domain for RE optimization.**Input:** Present iteration threshold ϵ, beam domain channel statistics $\Omega_{k}$, initial power allocation $\Lambda^{\left(0\right)}$,
**Output:** Power allocation matrix Λ 1: Initialization:ℓ=0; calculate $\eta^{\left(\ell \right)}$ with (20)
 2: **repeat**
 3:    Let ℓ←ℓ+1
 4:    Solve (19) with $\eta^{\left(\ell -1\right)}$ to obtain $\Lambda^{\left(\ell \right)}$
 5:    Calculate $\eta^{\left(\ell \right)}$ by (20)
 6: **until**
$|2\eta^{\left(\ell \right)}\sqrt{\min_{k}\;{\bar{R}}_{k}^{\left(\ell \right)}\left(\Lambda \right)}-{\left(\eta^{\left(\ell \right)}\right)}^{2}P\left(\Lambda \right)+\min_{k}\;\beta \frac{{\bar{R}}_{k}^{\left(\ell \right)}\left(\Lambda \right)}{P_{\mathrm{tot}}}|<\epsilon$
 7: Return $\Lambda =\Lambda^{\left(\ell \right)}$


## 4. Numerical Results

Through numerical analysis, the performance of the proposed RE optimization precoding is evaluated with statistical CSI available at the BS in massive MIMO multicast transmission. The main setup of the simulation parameters is listed below in Table 1 [15,29,32].

**Figure 1:**
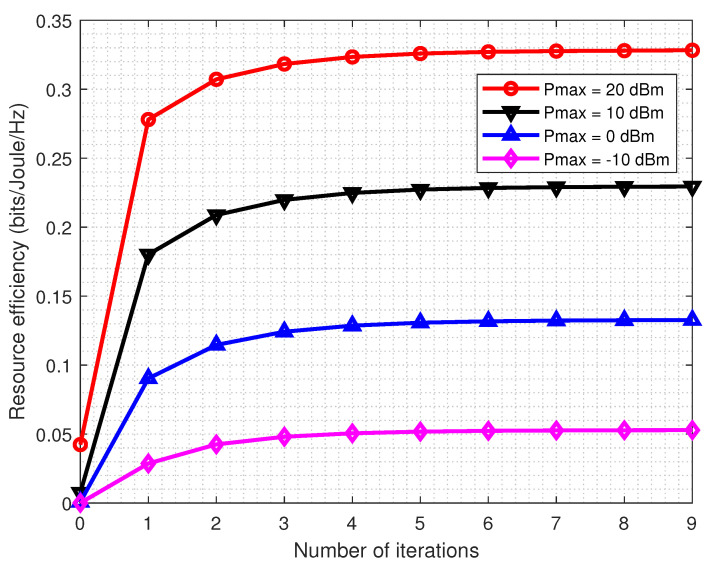
The convergence properties of Algorithm 1 for different multicast power budgets $P_{\max}$ with K=8.

In the first place, the convergence properties of the proposed power allocation algorithm versus the number of iterations under different multicast power budgets are evaluated. From Figure 1, it can be observed that the proposed algorithm tends to converge fast in just a few iterations for different multicast power budget $P_{\max}$ values. Besides, the result shows that in the typical transmit power budget region, Algorithm 1 can generate a non-decreasing sequence of RE values, which converges quickly. Specifically, in the high transmit power budget region, the system RE converges after only six iterations. As more iterations are needed to achieve the point of convergence for higher $P_{\max}$, Algorithm 1 converges slightly more slowly with $P_{\max}$ increasing.

**Figure 2:**
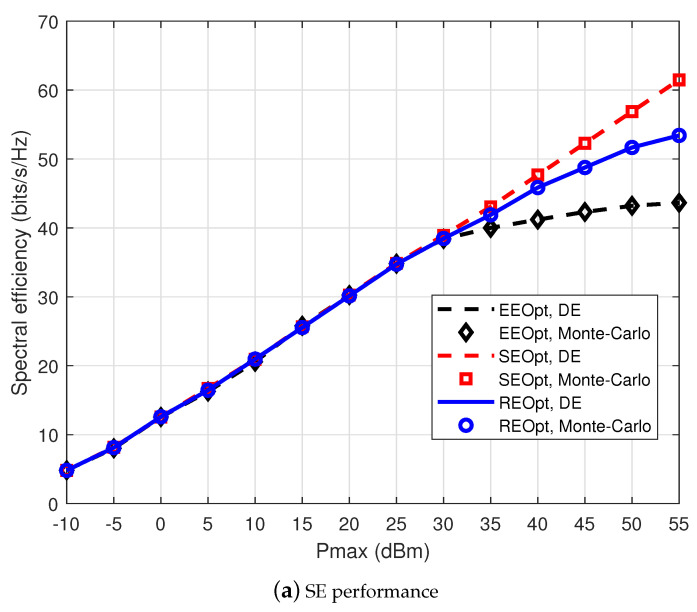

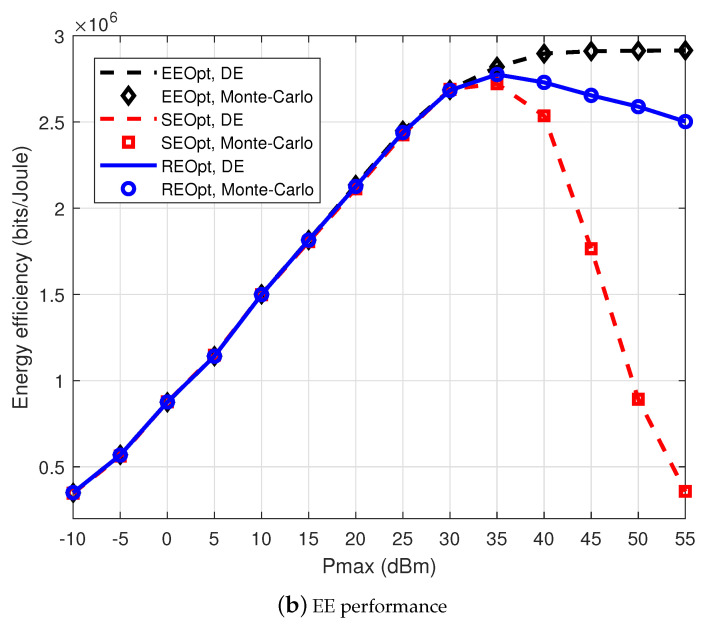
The comparison between the SE and EE performance versus different multicast power budgets $P_{\max}$. EEOpt, energy efficiency optimization; SEOpt, spectral efficiency optimization; DE, deterministic equivalent.

In order to prove the effectiveness of the approach proposed above for resource efficiency optimization (REOpt), we investigate the SE and EE performance versus different power budgets $P_{\max}$ of the three approaches in Figure 2. In the case of low power budgets, it is observed that the three approaches considered manifest nearly the same performance, and both SE and EE reach their optimization with $P_{\max}\leq 30\;\mathrm{dBm}$, which shows that multicast transmission could reach an approximate optimization in the case of low multicast power budgets. For comparison, besides the EE-SE tradeoff achieved by the proposed approach, the homologous performance of the spectral efficiency optimization (SEOpt) and energy efficiency optimization (EEOpt) methods is also plotted. Figure 2 proves that the REOpt method has the capacity to balance SE and EE, while the traditional SEOpt/EEOpt method just considers a single criterion. In the case of high multicast power budgets, the approach proposed above for REOpt obtains neither SE optimization nor EE optimization, but a balance between the SE and EE, which fits our target of the RE optimization design to tradeoff the SE and EE. Moreover, it can be observed that compared with the Monte Carlo approach, the result of the proposed DE approach is also considerably accurate. Meanwhile, the DE approach has better convergence properties. We can efficiently calculate the DE within several iterations; there is no need to adopt the Monte Carlo approach for exhaustive averaging; thereby, the time complexity of RE maximization problem is further reduced.

**Figure 3:**
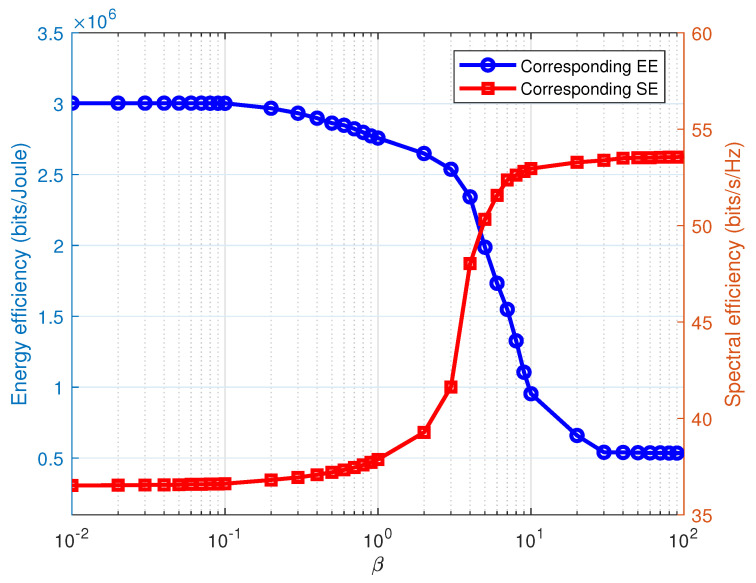
Effect of the weight factor β on the corresponding EE and SE.

**Figure 4:**
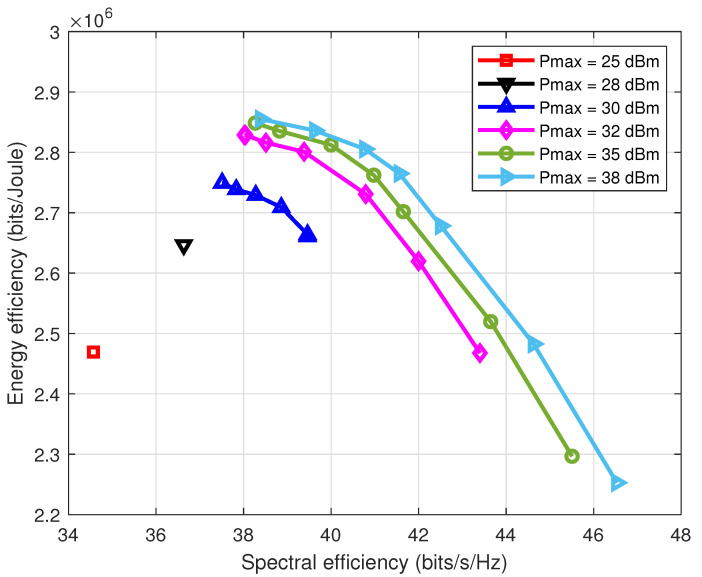
Tradeoff curve of EE and SE under different multicast power budgets.

By showing the corresponding system SE and EE with regard to multiple β values, Figure 3 illustrates the influencing degree of weight factor β. The result shows that increasing β will improve system SE, but reduce system EE, which is because the increasing β gives the SE a higher priority; thereby, more power is allocated to the SE maximization. In addition, if β→∞, the RE maximization method is reduced to maximizing system SE, and if β→0, it switches to the EE maximization method. We then present the tradeoff curve of the EE and SE in different multicast power budget regions in Figure 4 to further prove the performance of the proposed RE optimization method. It can be observed that the proposed Algorithm 1 begins to tradeoff the EE and SE when the multicast power budget $P_{\max}\geq 30\;\mathrm{dBm}$. In general, Figure 3 and Figure 4 demonstrate the capacity of the proposed RE optimization method to tradeoff the EE and SE by choosing an appropriate weighting factor β.

**Figure 5:**
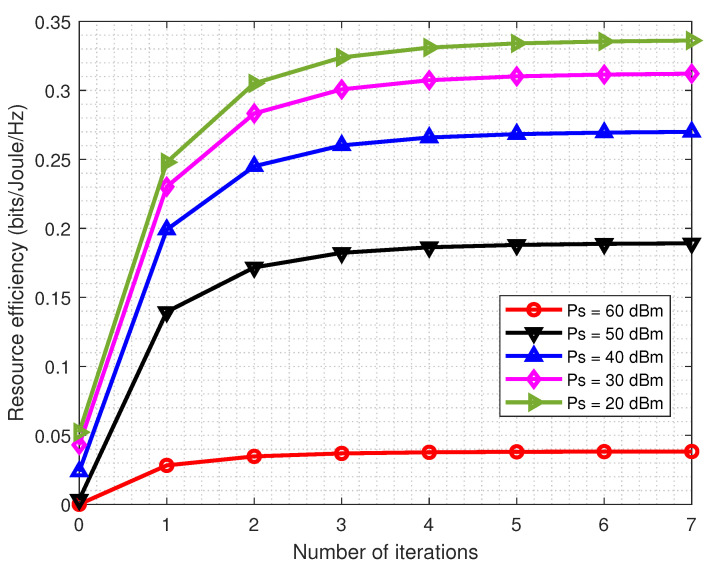
Convergence behavior of Algorithm 1 for different values of static power consumption $P_{s}$.

Finally, we evaluate the convergence behavior of Algorithm 1 for different values of static power consumption $P_{s}$ in Figure 5. As we can see, the RE decreases when the static power consumption $P_{s}$ increases because in the power consumption model we adopted in (8), the total circuit power consumption grows linearly with $P_{s}$.

## 5. Conclusions

To summarize, this paper researches the EE-SE tradeoff multicast precoding design in massive MIMO transmission with the BS only knowing statistical CSI. Firstly, the massive MIMO channel model in the beam domain is introduced. On this basis, we reveal the multicast signaling directions for RE optimization, which simplify the large-dimensional complex matrix-valued precoding design to a power allocation problem. Via exploiting the quadratic transform, we further propose an iterative power allocation algorithm that can ensure the global optimal solution. Besides, we adopt the theory of large-dimensional random matrices to deduce the DE of the design target. Finally, simulation results indicate that compared to the traditional approaches, the proposed RE optimization approach has a higher performance gain, especially under high multicast power budgets. Besides, in our proposed algorithm, the sub-problems are solved by the CVX optimization toolkit, which is an open-source tool and may suffer from uncertainty. In future research, a low-complexity iterative algorithm based on methods such as the water-filling scheme and subgradient method can be investigated.

## Figures and Tables

**Table 1:** Simulation parameters.

Parameter	Value
Propagation scene	Suburban macro
Model	3GPP SCM
Array topology	Uniform linear array (ULA)
Antenna spacing	Half wavelength
Transmission bandwidth	W= 10 MHz
Noise variance	$\sigma^{2}=-131$ dBm
Number of UTs	K=8
Number of UT antennas	$N_{k}=4\left(\forall k\right)$
Number of BS antennas	M=128
Amplifier drain efficiency	ζ=5
Path loss for each UT	−120 dB $\left(\forall k\right)$
Circuit power consumption	$P_{c}=30$ dBm per antenna
Static power consumption	$P_{s}=40$ dBm
